# Low level of tonic interferon signalling is associated with enhanced susceptibility to SARS-CoV-2 variants of concern in human lung organoids

**DOI:** 10.1080/22221751.2023.2276338

**Published:** 2023-11-16

**Authors:** Meaghan Flagg, Kerry Goldin, Lizzette Pérez-Pérez, Manmeet Singh, Brandi N. Williamson, Nathanael Pruett, Chuong D. Hoang, Emmie de Wit

**Affiliations:** a Laboratory of Virology, Division of Intramural Research, National Institute of Allergy and Infectious Diseases, National Institutes of Health, Hamilton, MT, USA; b Thoracic Surgery Branch, Division of Intramural Research, National Cancer Institute, National Institutes of Health, Bethesda, MD, USA

**Keywords:** SARS-CoV-2, variants of concern, tonic interferon, organoids, pathogenesis

## Abstract

There is tremendous heterogeneity in the severity of COVID-19 disease in the human population, and the mechanisms governing the development of severe disease remain incompletely understood. The emergence of SARS-CoV-2 variants of concern (VOC) Delta (B.1.617.2) and Omicron (B.1.1.529) further compounded this heterogeneity. Virus replication and host cell damage in the distal lung is often associated with severe clinical disease, making this an important site to consider when evaluating pathogenicity of SARS-CoV-2 VOCs. Using distal human lung organoids (hLOs) derived from multiple human donors, we compared the fitness and pathogenicity of SARS-CoV-2 VOC Delta and Omicron, along with an ancestral clade B variant D614G, and evaluated donor-dependent differences in susceptibility to infection. We observed substantial attenuation of Omicron in hLOs and demonstrated enhanced susceptibility to Omicron and D614G replication in hLOs from one donor. Transcriptomic analysis revealed that increased susceptibility to SARS-CoV-2 infection in these hLOs was associated with reduced tonic interferon signaling activity at baseline. We show that hLOs can be used to model heterogeneity of SARS-CoV-2 pathogenesis in humans, and propose that variability in tonic interferon signaling set point may impact susceptibility to SARS-CoV-2 VOCs and subsequent COVID-19 disease progression.

## Introduction

Comparison of SARS-CoV-2 Variant of Concern (VOC) pathogenesis in the human population is complicated by multiple factors, including variable pre-existing immunity due to vaccination and/or prior infection and the effect of increased caseloads on patient care [1]. To overcome these confounders, multiple studies have compared the pathogenesis of VOCs in animal models. Delta was reported to display enhanced pathogenicity in rodents, but this was not recapitulated in a non-human primate model [2–5]. In both rodents and non-human primates, Omicron-infected animals displayed reduced clinical disease signs, along with lower levels of virus replication, particularly in the lower respiratory tract [5–10].

While animal models have been useful for comparative pathogenesis studies, they present with several limitations. Rhesus macaques infected with SARS-CoV-2 generally display only mild respiratory disease [11, 12], preventing studies of severe disease pathogenesis. Syrian hamsters, while more prone to more severe disease following infection, have more significant sequence differences in critical spike-binding residues of ACE2 [13, 14], along with a respiratory tract structure that differs from humans and other larger mammals [15]. While ACE2 is much more similar between rhesus macaques and humans [16], differences in other host factors may still impact virus replication and when compounded with amino acid substitutions in VOCs, may lead to altered replication kinetics between species [17–19]. Evaluation of SARS-CoV-2 VOC in a human system representing the distal lung allows us to assess differences in pathogenicity in the absence of confounding factors.

Human lung organoids (hLOs) serve as a unique model of primary human respiratory epithelium [20, 21]. hLOs are three-dimensional multi-cellular structures that contain multiple lung epithelial cell types. Organoid models are advantageous compared to immortalized cell culture systems because they maintain innate immune pathway functionality, virus receptor expression, and cell type heterogeneity representative of their native tissue. The recent establishment of chemically-defined culture conditions to promote alveolar epithelial type II cell (AT2) expansion in organoids has opened avenues for studies of viral pathogenesis and host responses in distal lung epithelium [22–26], the part of the respiratory tract where virus infection can result in severe, often irreparable, damage. These organoids can be grown from adult stem cells isolated from donor lung tissue. Previous studies have demonstrated productive replication of SARS-CoV-2 in these cultures [22–28]. However, few studies have addressed variation in susceptibility and response to infection between organoids derived from different donors. In this study we conducted a comprehensive comparison of the fitness, pathogenicity, and host response to SARS-CoV-2 VOCs Delta, Omicron (BA.1 lineage), and an early clade B.1 isolate containing the D614G substitution in spike. We observed highly efficient replication of Delta in hLOs while Omicron was severely attenuated, in accordance with clinical reports and *in vivo* studies [5–10, 29–33]. Additionally, we demonstrated that productive replication occurred in the absence of significant epithelial cell death. Lastly, we observed enhanced susceptibility to infection in hLOs derived from one donor, which was accompanied by a distinct transcriptional response. Taken together, we validate the use of hLOs to model SARS-CoV-2 VOC fitness and emphasize their use to assess the effect of donor variation on pathogenesis.

## Results

### Generation of hLOs to model human lower respiratory tract epithelium

**Figure 1.**
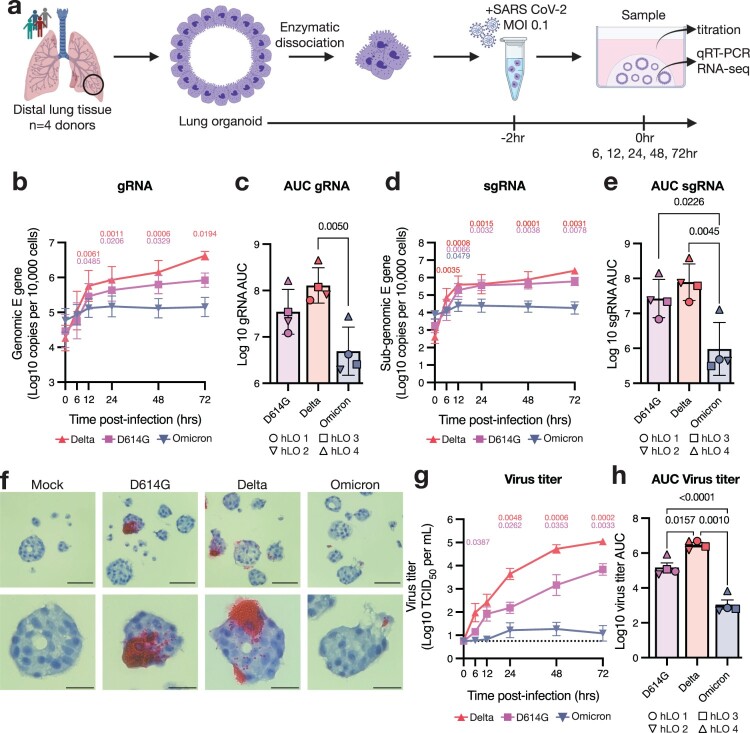
**SARS-CoV-2 VOCs Delta and D614G productively infect hLOs, while Omicron is attenuated.** (a) Schematic of hLO infection. Viral load in infected hLOs was measured by qRT-PCR for gRNA (b–c) or sgRNA (d-e) and averaged across hLOs from four donors. In each run, standard dilutions of counted RNA standards were run in parallel to calculate copy numbers in the samples. gRNA (c) or sgRNA (e) copy numbers over time were used to calculate area under the curve (AUC) relative to 0 h post-inoculation for each donor. (f) *In situ* hybridization against SARS-CoV-2 spike RNA was conducted in FFPE sections of hLOs 72 h post-inoculation. hLO 1 is shown. Scale bars denote 50 μm (upper panels) or 20 μm (lower panels). (g) Virus titers in the culture supernatant were quantified, and averaged across hLOs from four donors. Dotted line indicates lower limit of detection. (h) Virus titer over time was used to calculate AUC for each donor. Error bars denote mean +/− SEM. Statistical analysis was performed using two-way ANOVA with Dunnett’s post-test (b, d, g) or ordinary one-way ANOVA with Tukey’s post-test (c, e, h). P-values denote difference vs 0 h post-inoculation (b, d, g). *P*-values <0.05 are shown.

Distal lung tissue is comprised primarily of alveolar space lined by type I and type II alveolar cells (AT1 and AT2, respectively) which are major target cells of SARS-CoV-2 in the lower respiratory tract [34, 35]. To evaluate differences in fitness and pathogenicity of VOCs in physiologically relevant human lower respiratory tract epithelium, we generated lung organoid cultures derived from distal lung tissue sourced from four individual donors (Figure 1a, Supplementary Figure 1a). hLOs formed three-dimensional structures in culture with one or more lumens (Supplementary Figure 1b). hLOs expressed markers of AT2s including HTII-280 and surfactant protein C (SFTPC) (Supplementary Figure 1c−e). NK2 homeobox 1 (NKX2-1, also known as TTF-1) was expressed in hLOs, confirming their identity as distal lung epithelium (Supplementary Figure 1f). We also observed keratin 5 (KRT5)-expressing cells, indicating that hLOs also contain basal cells and/or transitional AT2 cells (Supplementary Figure 1f). hLOs expressed SARS-CoV-2 receptor ACE2 (Supplementary Figure 1g). Expression of advanced glycosylation endproduct (AGER) was not widely observed in hLOs (Supplementary Figure 1h), demonstrating a lack of differentiated AT1 cells, which are not generally present in 3D organoid cultures without the addition of specific differentiation medium [22] or subsequent 2D culture [23]. We did not detect significant expression of Club cell marker secretoglobin 1A1 (SCGB1A1) or multiciliated cell marker acetylated alpha tubulin (AcTub) in hLOs (Supplementary Figure 1i, j), indicating that our hLO cultures skewed towards alveolar, rather than airway epithelium as intended.

### SARS-CoV-2 Omicron BA.1 is attenuated in hLOs

We compared the fitness and pathogenicity of SARS-CoV-2 VOCs Delta (B.1.617.2), Omicron BA.1 (B.1.1.529), and an early clade B.1 isolate containing the D614G substitution in spike in hLOs derived from four donors (Figure 1a). Viral load as measured by genomic (gRNA) and sub-genomic (sgRNA) [36] RNA copy number increased over time in hLOs infected with the Delta and D614G variants (Figure 1b, d). We observed little to no increase in gRNA or sgRNA in Omicron-inoculated organoids. Over the course of the study, Delta- and D614G-infected hLOs produced significantly more viral RNA than Omicron-infected hLOs (Figure 1c, e). To visualize infected cells in hLOs we conducted *in situ* hybridization (ISH) against SARS-CoV-2 spike RNA. Delta- and D614G-infected hLOs contained ISH-positive cells that produced large amounts of viral RNA, while detection of ISH-positive cells in Omicron-inoculated hLOs was rare, and infected cells produced little RNA (Figure 1f). Lastly, we titered infectious virus released into the supernatant. hLOs infected with Delta produced significantly more infectious virus than D614G- or Omicron-infected hLOs. Omicron-inoculated hLOs produced little to no infectious virus (Figure 1g, h).

**Figure 2.**
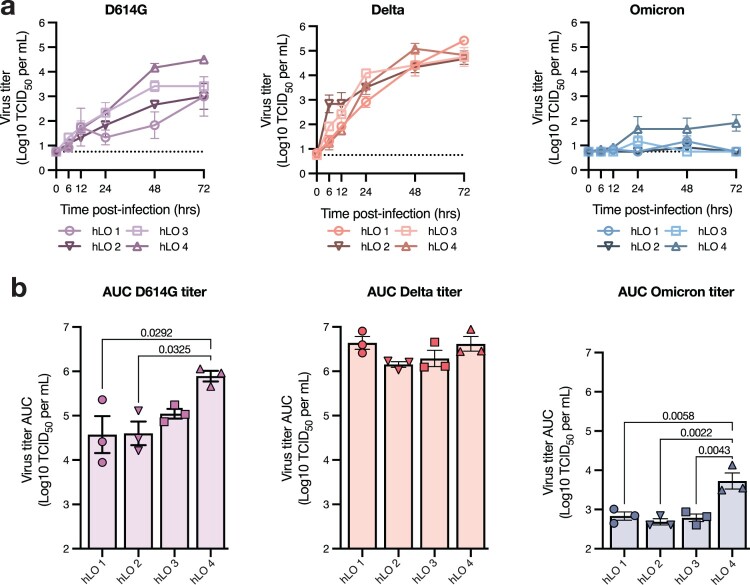
**Variable susceptibility to SARS-CoV-2 VOC infection in hLOs derived from different donors.** (a) Virus titers in culture supernatant were quantified. Dotted line indicates lower limit of detection. (b) Virus titer over time was used to calculate AUC for each donor. Mean +/− SEM of *n* = 3 replicates per donor hLO are shown. Statistical analysis was performed using one-way ANOVA with Tukey’s post-test (b). *P*-values <0.05 are shown.

We next analyzed hLOs from each donor separately to evaluate inter-individual variability in susceptibility to virus replication. Delta replicated equally well in hLOs from all donors (Figure 2a). Notably, we did observe production of infectious virus in Omicron-infected hLOs from one of the four donors (hLO 4) (Figure 2a). D614G also replicated to higher titers in hLOs from this individual (Figure 2a). Over the course of infection, D614G- and Omicron-infected organoids from hLO4 produced significantly more virus than hLOs from other donors, suggesting an enhanced susceptibility to SARS-CoV-2 infection (Figure 2b). We also analyzed sgRNA on an individual donor level to determine where the defect in Omicron replication was in the viral replication cycle. In hLO cultures where we did not detect Omicron replication (hLO 1–3), we observed a corresponding lack of an increase in sgRNA production with the exception of hLO 1 where we detected a slight increase at several timepoints (Supplementary Figure 2a, b) that did not amount to a significant difference in total viral load compared to other hLOs over the course of the study (Supplementary Figure 2c). This suggests that the poor replicative fitness of Omicron in hLOs from these donors is due to a defect early in viral replication. In agreement with virus titers, we observed significantly higher viral loads in hLO 4 over the course of infection (Supplementary Figure 2c), further suggesting enhanced permissiveness to infection in these hLOs.

### SARS-CoV-2 VOC replication is not cytotoxic in hLOs

**Figure 3.**
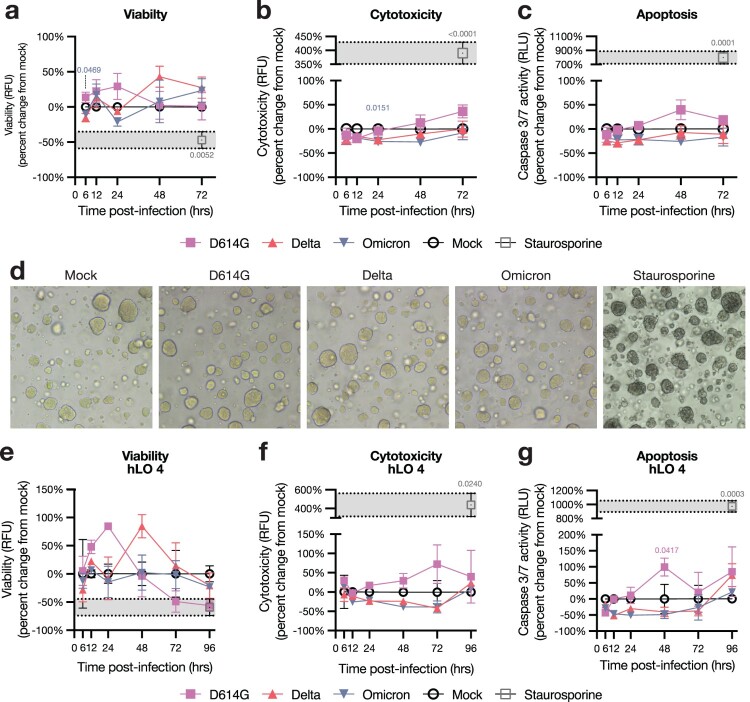
**hLOs survive infection with SARS-CoV-2.** hLO survival was analyzed over time after infection using the ApoTox-Glo triplex assay. hLO viability (a), cytotoxicity (b), and apoptosis (c) was blank-subtracted and normalized to hLO donor- and timepoint-matched mock-infected controls. Staurosporine was used as a positive control for cell death in these assays; the mean +/− SEM of staurosporine-treated samples is marked in gray shading. Mean +/− SEM of *n* = 4 hLO donors are shown. (d) Brightfield microscopy images of hLOs 72 h post-infection, or 6 h post-staurosporine treatment. (e) Viability, cytotoxicity, and apoptosis measurements for hLO 4, as in a–c. Mean +/ SEM of *n* = 3 hLO 4 replicates are shown. Statistical analysis was performed using two-way ANOVA with Dunnett’s post-test. *P*-values indicate difference vs mock-infected controls, values <0.05 are shown. RFU: relative fluorescence units, RLU: relative luminescence units.

Since pneumocyte death is observed in COVID-19 disease [37], we quantified the effects of SARS-CoV-2 infection on hLO survival. We utilized a triplex assay to measure viability, cytotoxicity, and caspase 3/7-dependent apoptosis in hLOs. We did not observe evidence of cell death in hLOs infected with any VOC, despite high levels of virus replication in Delta- and D614G-infected hLOs (Figure 3a–c). Organoid morphology was unchanged after infection (Figure 3d). We observed no alterations in organoid morphology or survival up to 8 days post-infection (data not shown). Cell death was not observed in hLOs from any individual donor, including hLO 4 which exhibited increased susceptibility to infection (Figure 3e–g).

### hLOs do not secrete pro-inflammatory cytokines in response to SARS-CoV-2 infection

To measure the innate immune response to SARS-CoV-2 infection, we conducted multiplex cytokine analysis on culture supernatants. We analyzed hLO culture supernatants for the presence of ten cytokines and chemokines commonly produced by epithelial cells in response to infection (TNF-α, IFN-α2a, IFN-β, IFN-λ1, IL-8, IL-1α, IL-1β, IL-6, IP-10, MCP-1). Although the levels of several analytes increased over time, we did not observe significant up-regulation of any cytokine compared to mock-infected controls (Supplementary Figure 3). Three cytokines (TNF-α, IFN-α2a, IFN-β) were below the limit of detection in the majority of samples. These data suggest either that hLOs did not mount a substantial inflammatory response to SARS CoV-2 VOCs, or that multiplex cytokine analysis was not sensitive enough to detect a response.

### Transcriptional analysis reveals significant donor-dependent variability

**Figure 4.**
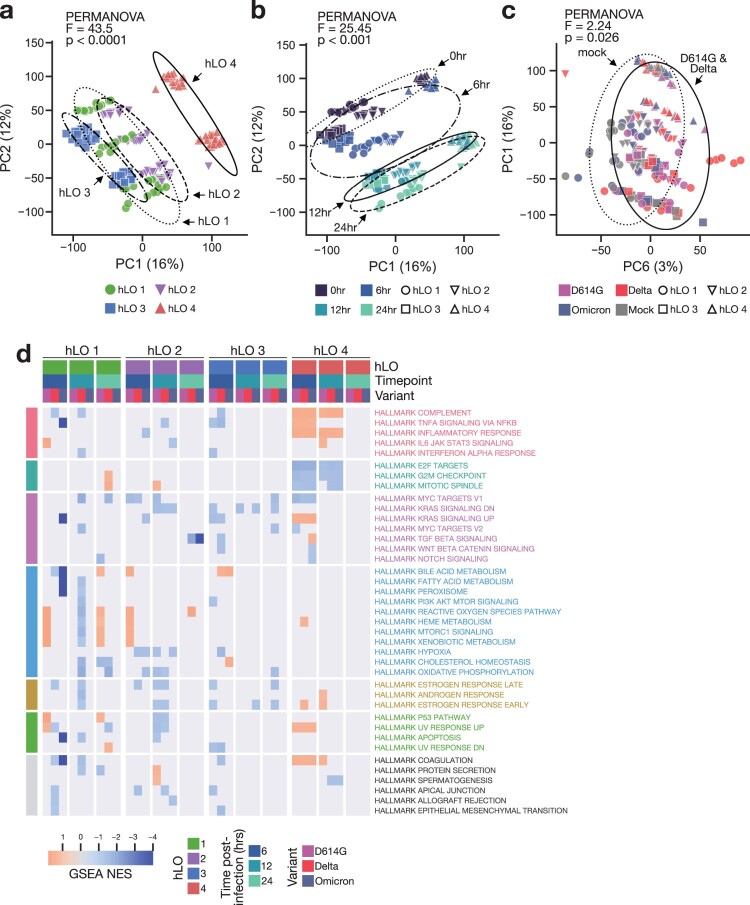
**Transcriptional response to SARS-CoV-2 infection is distinct in hLO 4.** (a–c) Principal component analysis (PCA) was conducted on VST-normalized gene counts. Axes are labelled with the principal component and the percentage of total variation captured by the component. Samples are plotted on principal components illustrating variation according to hLO donor (a), time post-infection (b), or infection status (c). Ellipses indicate two standard deviations from the group mean. PERMANOVA was computed on a weighted pairwise Minkowski distance matrix (see Supplementary Figure 4d). F-statistics and *p*-values are shown for the difference between hLO donor (a), time post-infection (b), or mock vs D614G- and Delta-infected samples (c). (d) Gene set enrichment analysis (GSEA) was conducted for each SARS-CoV-2 VOC vs mock-infected controls at 6, 12, and 24 h post-infection. Normalized enrichment scores (NES) for enriched pathways (false discovery rate (FDR) < 0.25) in two or more comparisons are shown. Cells where FDR > 0.25 are masked in gray. Statistical analysis was conducted using PERMANOVA (a–c). *P*-values < 0.05 are shown.

We employed total RNA sequencing (RNA-seq) of hLOs to analyze both the host response to infection within the first 24 h and any host-dependent variation in this response. We selected these timepoints to identify very early events in the host response to SARS-CoV-2, which have not previously been well-characterized. Whole-transcriptome principal component analysis (PCA) revealed significant transcriptional differences between hLOs derived from different donors, with hLO 4 clustering separately from the other three donors (Figure 4a). Samples also clearly clustered according to timepoint, even in the absence of infection (Figure 4b), demonstrating that hLOs undergo substantial transcriptional changes during the process of organoid re-formation. We conducted permutational multivariate analysis of variance (PERMANOVA) to statistically evaluate inter-group distances. The largest differences between samples were due to hLO origin, followed by time post-infection (Figure 4a, b). We did not detect significant separation by infection status (mock vs infected) or SARS-CoV-2 variant among all samples. However, we were able to detect a subtle, statistically significant, difference between D614G- and Delta-infected vs mock samples when we excluded Omicron-inoculated samples (Figure 4c). We suspect this is due to the inability of Omicron to productively replicate in hLOs from most donors, thereby failing to induce a significant transcriptional response.

We utilized differential gene expression analysis to identify specific transcriptional responses to infection. Since there were significant transcriptional differences between hLOs from the four donors, we modelled each hLO’s response to infection separately. We detected differentially expressed genes in response to infection in all hLOs, with hLO 4 showing the largest response (Supplementary Figure 4a). Interestingly, we observed little to no response to Omicron inoculation in hLOs 1, 2, and 3, suggesting that the lack of Omicron replication in these organoids is not due to induction of an innate immune response early following inoculation. hLO 4 showed a substantial response to Omicron infection (Supplementary Figure 4a), consistent with the productive replication observed in hLOs from this donor (Figure 2a). Accordingly, the number of significantly differentially expressed genes was positively correlated to virus titers measured from the same sample (Supplementary Figure 4b), suggesting a direct relationship between virus replication and the host transcriptional response.

We next compared the response to infection between hLOs from different donors. Surprisingly, we observed very little overlap in differentially expressed genes between hLOs from different donors (Supplementary Figure 4c). Attempts to model a common response across hLO donors to infection using DESeq2 were unsuccessful. For a more detailed analysis of relationships between samples, we computed a pairwise distance matrix, followed by hierarchical clustering (Supplementary Figure 4d). Samples from hLO 4 formed a separate cluster, while samples from hLO 1, 2, and 3 formed several sub-clusters according to early (0–6 h) or late (12–24 h) timepoints. We did not observe any clusters of infected samples belonging to multiple donors, further emphasizing a disparity in response to infection depending on hLO origin (Supplementary Figure 4d). We conducted gene set enrichment analysis (GSEA) to evaluate the response to infection in each hLO at the pathway level. Again, we observed different responses in hLOs from different donors (Figure 4d). We did not detect shared up- or down-regulation of pathways between variants or timepoints in hLO 1, 2, and 3, suggesting the lack of a cohesive response in cells from these hLOs. We did observe a concerted response in hLO 4, with an up-regulation of pro-inflammatory pathways and a down-regulation of pro-proliferative pathways in SARS-CoV-2-infected hLOs versus mock controls (Figure 4d). Together, these data demonstrate that hLO 4 mounted a response to SARS-CoV-2 infection that was distinct from other hLOs in our cohort.

### hLO 4 is transcriptionally distinct in the absence of infection

**Figure 5.**
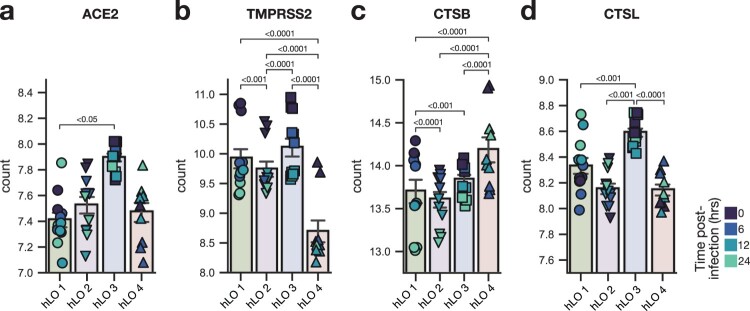
**Expression of SARS-CoV-2 entry factors in hLOs.** Normalized counts in mock-infected samples of ACE2 (a), TMPRSS2 (b), cathepsin B (c), and cathepsin L (d) are shown. Shading of points indicates time post-mock infection. Error bars denote grand mean +/ SEM of *n* = 12 replicates per hLO donor. Statistical analysis was conducted using Wald test with Benjamini-Hochberg adjustment for multiple test correction, as implemented in DESeq2. *P*-values < 0.05 are shown.

We compared differentially expressed genes between hLO 4 and the other three hLOs in mock-infected samples to identify baseline differences that may explain the enhanced susceptibility to infection in hLOs derived from this donor. We looked for differences in SARS CoV-2 entry factors ACE2 and TMPRSS2, along with cathepsin L (CTSL) and cathepsin B (CTSB) which are involved in the endosomal entry route, which is more efficiently utilized by Omicron compared to other VOCs [3]. ACE2 expression was largely similar between donors (Figure 5a), while TMPRSS2 expression was lower in hLO 4 (Figure 5b). CTSB was more highly expressed in hLO 4 (Figure 5c), and CTSL in hLO 3 (Figure 5d). Overall, we did not observe consistent patterns in SARS-CoV-2 entry factor expression that were likely to explain enhanced susceptibility to D614G and Omicron in hLO 4.

To determine if the differences in transcriptional response were related to the cellular composition of the hLOs, we analyzed expression of cell type marker genes. We selected genes that were reported to mark specific lung epithelial cell subsets in humans [38, 39]. We observed minor differences in expression of individual genes between donors (Supplementary Figure 5a), but not a concerted pattern of expression that would indicate substantial cell type composition differences in hLO 4. Additionally, when samples were hierarchically clustered according to expression of these genes, hLO 4 did not cluster separately (Supplementary Figure 5b), suggesting that the cell type composition of hLO 4 is unlikely to explain the different response observed in these hLOs.

### Reduced baseline IFN signaling may confer enhanced susceptibility to SARS-CoV-2 VOC infection

**Figure 6.**
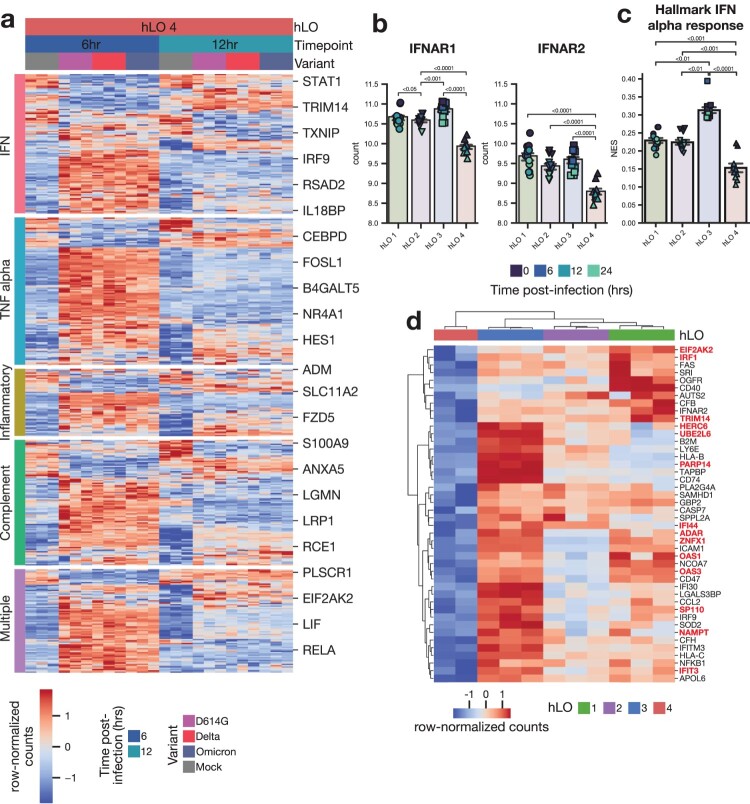
**Tonic IFN signaling is reduced in hLO 4.** (a) Differentially expressed genes (adjusted *p*-value < 0.1) in response to infection in hLO 4 were analyzed for their membership in the indicated MSigDB hallmark pro-inflammatory signaling pathways. Samples from 6 and 12 h post-infection are shown. (b) Normalized counts in mock-infected samples of IFNAR1 or IFNAR2 are shown. (c) ssGSEA normalized enrichment score (NES) for MSigDB Hallmark IFN alpha response pathway is shown in mock-infected samples. Shading of points indicates time post-mock infection. Error bars denote grand mean +/− SEM of *n* = 12 replicates per donor hLO. (d) Differentially expressed genes (adjusted *p*-value < 0.1) related to IFN response (MSigDB Hallmark IFN alpha response, Hallmark IFN gamma response) are shown comparing hLO 4 to other donors in the absence of infection. Genes involved in tonic IFN signaling are highlighted in red.

Since hLO 4 was more susceptible to SARS CoV-2 D614G and Omicron infection, and displayed significant transcriptional differences at baseline, we hypothesized that this susceptibility may be due to a deficiency in a component of the innate immune response. We looked for overlap in genes differentially expressed in this hLO in response to infection and known innate immune signaling pathways (Figure 6a). Surprisingly, we saw a robust induction of genes involved in IFN and other innate immune signaling pathways in response to infection, demonstrating that the innate immune response in hLO 4 was intact.

We next analyzed differentially expressed genes at baseline in mock-infected samples for overlap with innate immune signaling pathways. Expression of type I IFN receptors IFNAR1 and IFNAR2 was significantly lower in hLO 4 compared to hLOs from other donors (Figure 6b). Single sample gene set enrichment analysis (ssGSEA) revealed a corresponding reduction in type I IFN signaling activity (Figure 6c). We found numerous IFN-responsive genes were downregulated in hLO 4 compared to other donors at baseline (Figure 6d). Notably, many of these genes have been shown to be regulated specifically by tonic IFN signaling activity (Figure 6d) [40]. These data lead us to hypothesize that low levels of tonic IFN signaling prior to infection may leave the epithelium more susceptible to successful D614G and Omicron replication.

### SARS-CoV-2 D614G and Omicron are more susceptible to inhibition by type I IFN

Since in the context of low tonic IFN signaling in hLO 4 we detected increased replication of D614G and Omicron but not Delta, we reasoned that this could be due to differences in the sensitivity of the VOCs to inhibition by IFN. To test this we utilized A549 AAT cells, a lung adenocarcinoma cell line transduced to express ACE2 and TMPRSS2, since they support replication of all three VOCs (Supplementary Figure 6a). We pre-treated cells for 12 h prior to infection with escalating doses of IFNβ to induce different levels of baseline antiviral state, and analyzed the effect on subsequent virus replication. We measured the mRNA expression of three IFN-stimulated genes (ISG15, ISG20, IFITM3) after pre-treatment to confirm establishment of a dose-dependent antiviral state (Supplementary Figure 6b). Replication of SARS-CoV-2 D614G and Omicron in the presence of IFNβ was significantly more reduced compared to Delta, as evidenced by a ten-fold reduction in the IC90 value (Supplementary Figure 6c). The degree of sensitivity to IFN inhibition of each variant aligns with the selective replication advantage observed for D614G and Omicron in hLO 4, suggesting that low tonic IFN signaling activity could explain the enhanced replication of these variants in hLO 4.

## Discussion

Evaluation of SARS-CoV-2 VOC pathogenesis is critical to formulate appropriate public health responses. In this study, we show that viral fitness in hLOs is consistent with clinical reports and *in vivo* observations in animal models. We observed substantial attenuation of SARS-CoV-2 Omicron in hLOs, which is aligned with reports of reduced clinical disease in humans [29–33] and limited virus replication in the lower respiratory tract of infected rodents and non-human primates [5–10]. In contrast, SARS-CoV-2 Delta replicated with high efficiency in hLOs, consistent with increased transmissibility and disease severity in humans [30, 41–44]. Thus, hLO models can be used to bridge the gap between animal and epidemiological studies by facilitating analysis of viral fitness and pathogenicity in relevant primary human respiratory epithelium.

Interestingly, we did not observe host cell death in SARS-CoV-2-infected hLOs, despite high levels of virus replication with D614G and Delta. Several studies reported apoptosis of AT2 cells in SARS-CoV-2-infected organoids [22, 27], while others did not observe or specifically assess host cell survival [23, 24, 26]. These studies were all conducted with the SARS-CoV-2 WA-1 isolate, which may partially explain differences in reported AT2 cell apoptosis. We utilized a robust and sensitive assay to detect changes in host cell survival, including both apoptotic and non-apoptotic cell death, at multiple time points post-infection, and observed no evidence of cell death. Interestingly, there are several reports that SARS-CoV-2 infection can induce cell death when AT2 cells were co-cultured alongside other cell types, including differentiated AT1, proximal airway cells, or macrophages, and this is associated with a strong pro-inflammatory response [25, 45]. In line with these observations, hLOs in our study did not contain these cell types, and we did not detect pro-inflammatory cytokines in hLO supernatant or a clear pro-inflammatory response on the transcriptional level in three of four donor hLOs. This suggests that SARS-CoV-2-associated AT2 cell death may not be AT2 cell-intrinsic, and further studies involving co-culture of additional epithelial and non-epithelial cells are necessary to elucidate the specific mechanisms of cellular crosstalk between the diverse cell populations in the lung that contribute to epithelial death *in vivo*.

There is tremendous variability in the severity of COVID-19 disease in the human population. Adult stem cell-derived hLOs from multiple donors may serve as a unique tool to model this heterogeneity. In this study, we observed enhanced virus replication of SARS-CoV-2 D614G and Omicron in hLOs from one out of four donors (hLO 4). Interestingly, while hLOs from this individual were capable of mounting an innate immune response, they displayed lower levels of type I IFN signaling activity at baseline. Constitutive low-level type I IFN production and signaling through IFNARs is known to occur in the absence of infection [46], and was shown to play a protective role against influenza A virus infection in mice [47, 48]. A recent study demonstrated that primary nasal epithelial cells isolated from a donor with intrinsic high levels of IFNλ production were resistant to SARS-CoV-2 infection [49]. Since tonic IFN signaling has been shown to occur through type I IFN receptors [47], we hypothesize that the reduced expression of IFNAR1 and IFNAR2 in hLO 4 is responsible for the lower expression levels of IFN-stimulated genes (ISGs) at baseline. Our study suggests that a hLO donor-dependent lack of tonic IFN signaling is associated with increased virus replication. Interestingly, this enhanced susceptibility differed between SARS-CoV-2 VOCs, since increased virus replication was not observed in Delta-infected hLOs from donor 4. We attribute this to the differential susceptibility of SARS-CoV-2 VOCs to inhibition by IFN. Delta is reported to possess an enhanced ability to evade innate immune control [50, 51], and we observed reduced sensitivity of Delta to IFN inhibition compared to D614G and Omicron, thus making Delta less affected by the host tonic IFN set point. An efficient type I IFN response is essential to control SARS-CoV-2 replication and prevent development of severe COVID-19. Genetic deficiencies in components of the type I IFN signaling pathway, including IFNAR1 and IFNAR2, along with downstream kinases JAK1 and TYK2, are associated with development of severe COVID-19 [52, 53] while expression of certain alleles of IFN-induced dsRNA sensor OAS1 correlate with reduced disease severity [54, 55]. Several of these genes, specifically IFNAR1, IFNAR2, JAK1, and TYK2, are involved in the control of tonic IFN signaling. The impact of variation in tonic IFN signaling set points on the severity of COVID-19 has not been explored. Given the established importance of the post-infection IFN response, it is plausible that individuals with a low tonic IFN set point may be at greater risk of severe COVID-19.

This study has several limitations. Firstly, COVID-19 pathogenesis is complex and involves crosstalk between numerous cell types, including epithelial and immune cells. The hLOs used in this study contain only epithelial cells and are therefore not capable of fully modelling this complexity. Future studies should explore co-culture of immune and epithelial cells to probe cell–cell interactions that contribute to pathogenesis. A second limitation is the small number of hLO donors included in this study. Due to the substantial differences we observed between donors, a larger cohort is necessary to more accurately represent the variation in host responses to SARS-CoV-2 infection. Lastly, while we observed reduced tonic IFN signalling and enhanced susceptibility to SARS-CoV-2 infection in hLO 4, we cannot definitively conclude whether this would contribute to more severe disease in this donor. Future studies in larger cohorts should explore the mechanisms underlying the differential set points of tonic IFN signaling, and incorporate clinical outcome data to determine whether this influences COVID-19 severity *in vivo*.

Together, our study demonstrates that adult stem cell-derived hLOs are a unique tool to accurately model SARS-CoV-2 VOC fitness, donor-dependent variation in susceptibility to SARS-CoV-2 infection, and the subsequent host response to this infection. As such, exploration of the mechanisms behind this donor-dependent variability can reveal novel insights in the heterogeneity of COVID-19 pathogenesis in humans.

## Methods

### Study design

The objective of this study was to compare the fitness and pathogenicity of SARS-CoV-2 VOCs in lung organoids from multiple human donors. We inoculated hLOs derived from four human donors with SARS-CoV-2 D614G, Delta, and Omicron, and compared virus replication, host cell survival, and host responses over time between variants and hLO donors. For all experiments we included three replicates of each hLO culture to distinguish experimental from donor-to-donor variation. For hLO viability, multiplex cytokine, and transcriptional analysis, infected samples were compared to timepoint- and hLO donor-matched mock-infected controls. For multiplex cytokine and transcriptional analyses, samples were randomized to avoid batch effects. Outlier exclusion criteria for multiplex cytokine and transcriptional analyses are described below.

### Ethics and biosafety statement

De-identified human lung tissue samples were collected in accordance with Institutional Review Board-approved protocols at the NIH Clinical Center. The Institutional Biosafety Committee (IBC) approved work with SARS-CoV-2 under Biosafety Level 3 conditions. Sample inactivation was conducted according to IBC-approved standard operating procedures for removal of specimens from high containment.

### Cells and viruses

Vero E6 cells (R. Baric, University of North Carolina at Chapel Hill) were maintained in DMEM with 10% fetal bovine serum (FBS, Gibco), 1 mM L-glutamine (Gibco), 50 U/mL penicillin (Gibco), and 50 μg/mL streptomycin (Gibco) (DMEM-10). Vero E6-TMPRSS2-T2A-ACE2 cells (BEI resources) were maintained in DMEM-10. HA-R-Spondin1-Fc 293 T cells (R&D Systems) were maintained in DMEM-10 supplemented with 300 μg/mL Zeocin (InvivoGen). A549 cells transduced with ACE2 and TMPRSS2 (A549 AAT) [56] were maintained in DMEM-10. Selection with 200 μg/mL Hygromycin B and 50 μg/mL Geneticin was performed every five passages.

D614G (SARS-CoV-2/human/USA/RML-7/2020; GISAID EPI_ISL_591054) was grown from a nasopharyngeal swab obtained on 19 July 2020 [4]. Delta (hCoV-19/USA/MD-HP05647/2021; GISAID EPI_ISL_2331496) was obtained from A. Pekosz, Johns Hopkins Bloomberg School of Public Health [5]. Omicron (BA.1, hCoV-19/USA/GAEHC-2811C/2021, GISAID EPI_ISL_7171744) was obtained from M. Suthar, Emory University School of Medicine [5]. Viruses were propagated in Vero E6 cells in media as above, except with 2% FBS (DMEM-2). Virus stocks were sequenced, and no mutations were detected. All cells and virus stocks in this study tested negative for mycoplasma.

### Production of R-Spondin1 conditioned media

HA-R-Spondin1-Fc 293 T cells were used to produce R-Spondin1 conditioned media according to the manufacturer’s recommended protocol. Briefly, cells were seeded in T-150 flasks and cultured in DMEM-10 supplemented with 300 μg/mL Zeocin until 90% confluent. Medium was changed to DMEM-10 without selection antibiotics for 24 h. Medium was then replaced with 25 mL Advanced DMEM/F12 (Thermo Fisher Scientific) supplemented with Glutamax (Thermo Fisher Scientific), (ADF). Conditioned ADF was then collected every 24 h and pooled for seven days. Media was centrifuged at 2000×g for 10 min to remove cellular debris, then passed through a 0.22 μm filter. Aliquots of conditioned media were stored at −20°C.

### Human lung tissue dissociation

Adult distal lung tissue samples were obtained from four individuals (Supplementary Table 1) undergoing lung resection surgery at the NIH Clinical Center. Specimen collection followed our institutional review board (IRB)-approved protocols. De-identified normal lung tissue was collected, immediately placed in transport media (Supplementary Table 2), and shipped on cold packs overnight for processing. A single cell suspension was generated as follows: tissue was finely minced using scissors, then transferred to a 15 mL conical tube containing transport media supplemented with 0.35 mg/mL Liberase TM (Millipore Sigma). Tissue was incubated at 37°C for 45 min, with agitation every 15 min. Cells were then mechanically dissociated using an 18-gauge needle with syringe. Cells were centrifuged and resuspended in ACK lysis buffer (Thermo Fisher Scientific) for 90 s to remove red blood cells. The reaction was quenched with transport media supplemented with 5% FBS and filtered through a 100 μm cell strainer. Epithelial cells were enriched via magnetic-activated cell sorting (MACS). Negative selection was performed by MACS according to the manufacturer’s instructions using anti-CD45 (Miltenyi), anti-CD31 (Miltenyi), and anti-Fibroblast (Miltenyi) microbeads. The negative fraction, containing epithelial cells, was then seeded for organoid culture.

### hLO culture

Epithelial cells were cultured in a three-dimensional extracellular matrix (ECM) containing 66% (vol/vol) phenol red-free, LDEV-free, growth factor-reduced Matrigel (Corning) supplemented with 33% (vol/vol) lung organoid culture medium (Supplementary Table 2). Cells were seeded at 1000–2500 cells per μL. For infection experiments, cells were seeded at 2500 cells per μL. ECM was solidified for 30 min at 37°C, after which lung organoid culture medium was overlaid. Culture medium was changed weekly. Organoids were passaged every 3–4 weeks. ECM domes were dislodged in phosphate-buffered saline (PBS) with vigorous pipetting. Cells were centrifuged and residual ECM was removed. Cells were then resuspended in TrypLE Select (Thermo Fisher Scientific) and incubated for 10 min at 37°C with shaking. TrypLE was removed, and cells were mechanically dissociated in a small volume of culture medium using a narrow-mouth pipet tip. Organoids were able to be cultured for upwards of ten passages. hLOs were used for experiments between passages three and five.

### Flow cytometry

Uninfected hLOs were dissociated into single cells using TrypLE as described above. hLO cells were incubated with primary antibodies (EpCAM and HTII-280, Supplementary Table 3) along with LIVE/DEAD fixable Far Red Dead Cell Stain (Thermo Fisher Scientific) diluted in PBS with 1% FBS for 30 min at 4°C. Cells were washed in PBS with 1% FBS, then incubated in secondary antibody (anti-mouse IgM, Supplementary Table 3) diluted in PBS with 1% FBS for 30 min at 4°C. Cells were washed in PBS with 1% FBS, then fixed in 2% paraformaldehyde for 1 h at 4°C before analysis using a FACSymphony (BD Biosciences). Data were analyzed with FlowJo (v10.6.1). Gates were set using fluorescence minus one (FMO) controls.

### Whole-mount immunostaining of hLOs

hLOs were cultured as described above in chambered coverslips (Ibidi). hLOs were fixed in 10% neutral buffered formalin for 24 h at 4°C, washed in PBS, and permeabilized and blocked with serum-free protein block (Agilent/Dako) supplemented with 0.5% Triton X-100 for four hours at room temperature. hLOs were incubated with primary antibodies (NKX2.1, HTII-280, KRT5, Supplementary Table 3) overnight at 4°C. hLOs were washed three times in PBS with 0.05% Tween-20 (PBST), then incubated with secondary antibodies (Supplementary Table 3) overnight at 4°C. hLOs were washed three times in PBST, then incubated with NucBlue fixed cell ReadyProbes (Thermo Fisher Scientific) for 10 min at room temperature. Cells were washed three times in PBS, then imaged using a Zeiss LSM 880 confocal microscope.

### Immunostaining of formalin-fixed paraffin-embedded (FFPE) hLOs

hLOs were fixed in 10% neutral buffered formalin for 24 h at 4°C, washed with 70% ethanol, and suspended in Histogel (Thermo Fisher Scientific) before dehydration and embedding in paraffin. Blocks were sectioned at 5 μm. Heat-induced epitope retrieval was performed using the TintoRetriever (Bio SB) for 10 min at high pressure (110–120°C) in either Borg Decloaker (Biocare Medical), or Diva Decloaker (Biocare Medical). Sections were washed with PBS and blocked with serum-free protein block for 1 h at room temperature. Primary antibodies (SftpC, ACE2, AGER, SCGB1A1, AcTub; Supplementary Table 3) were applied overnight at 4°C. Sections were washed three times in PBST, then incubated with secondary antibodies (Supplementary Table 3) for two hours at room temperature. hLOs were washed three times in PBST, then incubated with NucBlue fixed cell ReadyProbes for 10 min at room temperature. Sections were washed in PBS, then coverslips were mounted using ProLong Gold Antifade reagent (Thermo Fisher Scientific). Images were acquired using an Echo Revolve fluorescence microscope.

### RNA in situ hybridization

FFPE sections of SARS-CoV-2-infected hLOs were generated as described above. RNAscope VS Universal AP assay (Advanced Cell Diagnostics) was used to detect SARS-CoV-2 spike RNA using probe 848569 (Advanced Cell Diagnostics). The semi-automated assay was performed according to the manufacturer’s instructions with the following modifications: manual pre-treatment of slides was done by boiling in 1x RNAscope target retrieval reagent (Advanced Cell Diagnostics) at 100°C for 10 min followed by washing in distilled water. Protease treatment was reduced to 8 min. Slides were mounted with EcoMount (Biocare Medical).

### Infection of hLOs with SARS-CoV-2

hLOs were enzymatically and mechanically dissociated into single cells as described above. Cells were resuspended in transport media (Supplementary Table 2) and incubated with SARS-CoV-2 at MOI 0.1 for two hours at 37°C on a rotator. Cells were washed once in PBS to remove unbound virus, then seeded in 48-well plates (Genesee Scientific) in 10 μL ECM droplets at 2500 cells per μL, as described above. ECM was solidified for 30 min at 37°C, after which 500 μL lung organoid culture medium was overlaid and sampling began. Culture media was collected for virus titration and multiplex cytokine analysis, after which hLOs were lysed in Buffer RLT (Qiagen) for qRT-PCR and transcriptomic analysis.

### Virus titration

Virus titration was performed by endpoint titration in Vero E6-TMPRSS2-T2A-ACE2 cells. Cells were inoculated with ten-fold serial dilutions of hLO culture supernatant in DMEM-2. Plates were centrifuged for 30 min at 1000 rpm at room temperature, followed by incubation for 30 min at 37°C and 5% CO_2_. The inoculum was removed, cells were washed once with PBS, and media was replaced with 100μL DMEM-2. After six days, cytopathic effect was scored and the TCID_50_ was calculated.

### RNA isolation and qRT-PCR

RNA was extracted from hLOs or A549 AAT cells lysed in Buffer RLT buffer (Qiagen) using the RNeasy Mini Kit (Qiagen) according to the manufacturer’s instructions. 5 or 2 μL (A549 AAT) of RNA was used in a one-step qRT-PCR reaction using the QuantiNova Probe RT–PCR Kit (Qiagen) to detect gRNA [57, 58] (E) and sgRNA [36] (E) gene copies. In each run, standard dilutions of counted RNA standards were run in parallel, to calculate copy numbers in the samples. For quantification of host transcripts, cDNA was prepared using the SuperScript IV VILO kit with EZDNase (Thermo Fisher Scientific) according to the manufacturer’s instructions. qPCR was performed on a QuantStudio 6 instrument (Thermo Fisher Scientific) using the TaqMan Fast Advanced master mix (Thermo Fisher Scientific). Relative quantification analysis was conducted using GAPDH and ACTB as endogenous controls. FAM/ZEN/IowaBlackFQ-labelled probes (Integrated DNA Technologies) were used. Primer sequences are provided in Supplementary Table 4.

### Organoid survival assay

hLOs were seeded in black-walled 96-well microplates after infection at 2500 cells per μL in Matrigel as described above. Survival post-infection was measured using the ApoTox-Glo Triplex assay (Promega) according to the manufacturer’s instructions. Prior to final sampling at 72 h post-infection, hLOs were treated with 2.5 μM staurosporine (Selleck Chemicals) for 24 h as a positive control for apoptotic cell death. Data for each assay were blank subtracted to calculate relative fluorescence or luminescence units, then normalized to hLO donor- and timepoint-matched mock-infected controls.

### Multiplex cytokine analysis

Infected hLO supernatant samples were analyzed for the presence of TNFα, IFN-α2a, IFN-β, IFN-λ1, IL-1β, IL-1α, IL-8, IL-6, IP-10, and MCP-1 using the U-PLEX custom assay (Meso Scale Diagnostics) according to the manufacturer’s instructions. Samples were run in technical duplicate and randomized across plates to mitigate batch effects. Samples were inactivated via 2MRad gamma-irradiation before analysis [59]. Analyte concentrations and upper and lower limits of detection were determined using Discovery Workbench software (Meso Scale Diagnostics). Samples that fell outside of the detection range were replaced with the corresponding upper or lower limit of detection value. Data were analyzed using Python (version 3.8.15). Technical outliers were excluded if the coefficient of variation (CV) between technical replicates was >20% and the sample concentration differed from the mean of all samples by more than 2.5 times the standard deviation. Infected samples were compared against mock-infected controls collected at the last timepoint.

### Bulk RNA-sequencing and gene expression analysis

Sequencing libraries were generated from RNA extracted as above using the SMARTer Stranded Total RNA-Seq Kit v3 – Pico Input Mammalian (Takara). Prior to library construction, genomic DNA was removed by re-extraction of RNA samples using the AllPrep DNA/RNA 96-well system (Qiagen) including an additional on-column DNase I treatment. RNA integrity was assessed using the Agilent 2100 Bioanalyzer using RNA 6000 Pico kit (Agilent Technologies). RNA was quantified using fluorescence assay Quant-iT RiboGreen RNA Assay (Thermo Fisher Scientific), and measured using Tecan Spark (Tecan Group Ltd.). 100 bp paired-end sequencing was performed using NovaSeq (Illumina) to a mean depth of 52 million reads per sample. Adapter sequences were trimmed and low-quality reads were removed using Cutadapt. FastQC was used to evaluate sequence quality and adapter content before and after trimming. Reads were mapped to a combined human (hg38, Gencode v39) and SARS-CoV-2 (wuhCor1, UCSC) reference genome using STAR. One sample was excluded due to poor alignment rate. Expected gene counts and transcripts per million (TPM) were obtained with RSEM. Genes absent in >90% of samples were excluded from downstream analyses. Gene-level counts are contained in Data file S1. Gene counts were normalized using the variance-stabilizing transformation (VST) implemented in DESeq2. Differential gene expression analysis was performed on non-normalized rounded expected counts using DESeq2. Gene set enrichment analysis (GSEA) was performed on ranked gene lists using the gseaprerank function implemented in gseapy. Genes were ranked by multiplying the log2 fold change by the −log10 of the adjusted *p*-value (pi score), calculated in DESeq2. Single-sample GSEA (ssGSEA) was performed on TPM data using the ssGSEA function implemented in gseapy.

Principal component analysis (PCA) was computed on scaled VST-normalized counts using scikit-learn. A pairwise distance matrix was calculated on PCA-transformed data using the weighted Minkowski metric, with the percentage of variance explained by each PC used as weights. The distance matrix was calculated using the pdist function implemented in scipy. Hierarchical clustering was conducted using the linkage function implemented in scipy.

### VOC IFN sensitivity assay

A549 AAT cells were treated with escalating doses of recombinant human IFNβ (Peprotech) for 12 h. Medium was replaced with DMEM-10 not containing IFNβ and cells were infected with SARS-CoV-2 VOCs at MOI 0.1 for one hour at 37°C and 5% CO_2_. Cells were washed, medium was replaced, and cells were incubated for 24 h at 37°C and 5% CO_2_. Host IFN-stimulated genes and SARS-CoV-2 sgRNA were quantified by q-RT–PCR. Normalized sgRNA copies were calculated by subtracting the initial input (copies present at 0hr post-infection), followed by normalizing to the percentage of copies present in untreated (no IFNβ) control samples. Five-parameter logistic regression curves were fit, IC90 was calculated, and data were plotted on a modified symmetrical log scale (asinh, implemented in Matplotlib v3.8).

### Statistical analysis and software

One- and two-way ANOVA and associated post-hoc tests were conducted using GraphPad Prism (v9.3.1). Flow cytometry data was analyzed using FlowJo (v10.6.1). Additional analyses were performed using Python (v3.8.15) and R (v4.1.3). Wald tests comparing gene expression were carried out using DESeq2, using the Benjamini-Hochberg multiple test correction. Permutational analysis of variance (PERMANOVA) was used to compare pairwise distances between sample groups. This was calculated using the permanova function implemented in scikit-bio. For IFN sensitivity comparisons five-parameter logistic regression curves were fit to normalized data with scipy (v1.11.1) and used to calculate IC90 values [60]. A *p*-value <0.05 was considered significant. Graphs were generated using GraphPad Prism, Matplotlib, or Seaborn.

## Supplementary Material

data_file_S1_expected_counts

Flagg_et_al_SARS2_VOC_in_hLOs_supplemental_revised_clean_version

## Data Availability

Data included in this manuscript have been deposited in Figshare at https://doi.org/10.6084/m9.figshare.22266313. Flow cytometry data are available at http://flowrepository.org/id/FR-FCM-Z65R. RNA sequence data sharing is restricted by Informed Consent; gene counts can be found in supplementary data file 1.
