# Supplementary material for: Low level of tonic interferon signalling is associated with enhanced susceptibility to SARS-CoV-2 variants of concern in human lung organoids
Source: Emerg Microbes Infect. 2023 Nov 16;12(2):2276338. doi: 10.1080/22221751.2023.2276338 (PMC10732190; doi:10.1080/22221751.2023.2276338)
Supplement: Flagg_et_al_SARS2_VOC_in_hLOs_supplemental_revised_clean_version [file TEMI_A_2276338_SM3870.docx]

Supplementary Materials for

**Low level of tonic interferon signaling is associated with enhanced susceptibility to SARS-CoV-2 variants of concern in human lung organoids**

Meaghan Flagg, Kerry Goldin, Lizzette Pérez-Pérez, Manmeet Singh, Brandi N. Williamson, Nathanael Pruett, Chuong D. Hoang, and Emmie de Wit^*^

*Corresponding author. Email: emmie.dewit@nih.gov

This file includes:

Supplementary table 1. Characteristics of hLO donors.

Supplementary table 2. hLO culture media composition.

Supplementary table 3. Antibodies used in this study

Supplementary table 4. Primer sequences used for q-RT-PCR

Supplementary figure 1. Characterization of hLOs.

Supplementary figure 2. Variable viral load in hLOs derived from different donors.

Supplementary figure 3. hLOs do not secrete cytokines in response to SARS-CoV-2 infection.

Supplementary figure 4. Divergent transcriptional responses to SARS-CoV-2 infection in hLOs from different donors.

Supplementary figure 5. Expression of canonical lung epithelial cell markers in hLOs.

Supplementary figure 6. IFN sensitivity of SARS CoV-2 VOCs

Other Supplementary Material for this manuscript includes the following:

Supplementary data 1. Gene counts and associated metadata.

**Supplementary table 1. Characteristics of hLO donors.**

| **Donor** | **Sex** | **Age (years)** |
| --- | --- | --- |
| hLO 1 | M | 53 |
| hLO 2 | F | 76 |
| hLO 3 | M | 67 |
| hLO 4 | M | 76 |

**Supplementary table 2. hLO culture media composition.**

| **Reagent** | | **Concentration** | | | **Vendor** | | **Catalog number** | | | |
| --- | --- | --- | --- | --- | --- | --- | --- | --- | --- | --- |
| *Transport Media* | | | |  | |  | | |  | |
|  | Advanced DMEM/F12 | |  | | Thermo Fisher Scientific | | | 12634028 | |  |
|  | HEPES buffer | | 10mM | |  | | |  | |  |
|  | Glutamax | | 1X | | Thermo Fisher Scientific | | | 35050061 | |  |
|  | Primocin | | 100ug/mL | | Invivogen | | | ant-pm-1 | |  |
|  | Y-27632 2HCl | | 10uM | | Selleck Chemicals | | | S1049 | |  |
| *Lung organoid culture media* | | | |  | |  | | |  | |
|  | Advanced DMEM/F12 | |  | | Thermo Fisher Scientific | | | 12634028 | |  |
|  | HEPES buffer | | 10mM | |  | | |  | |  |
|  | Glutamax | | 1X | | Thermo Fisher Scientific | | | 35050061 | |  |
|  | Primocin | | 100ug/mL | | Invivogen | | | ant-pm-1 | |  |
|  | R-Spondin1 conditioned media | | 10% | | produced in-house | | |  | |  |
|  | B-27 | | 1X | | Thermo Fisher Scientific | | | 17504044 | |  |
|  | Nicotinamide | | 10mM | | Millipore Sigma | | | N0636-100G | |  |
|  | N-acetyl-cysteine | | 1mM | | Millipore Sigma | | | A9165-5G | |  |
|  | Y-27632 2HCl | | 10uM | | Selleck Chemicals | | | S1049 | |  |
|  | recombinant human noggin | | 100ng/mL | | Peprotech | | | 120-10C | |  |
|  | recombinant human EGF | | 50ng/mL | | Peprotech | | | AF-100-15 | |  |
|  | recombinant human FGF-7 | | 100ng/mL | | Peprotech | | | 100-19 | |  |
|  | recombinant human FGF-10 | | 100ng/mL | | Peprotech | | | 100-26 | |  |
|  | SB431542 | | 10uM | | Selleck Chemicals | | | S1067 | |  |
|  | CHIR-99021 | | 3uM | | Selleck Chemicals | | | S1263 | |  |

**Supplementary table 3. List of antibodies used for immunofluorescence (IF) and flow cytometry (FC)**

| **Target** | **Host species** | **Fluorophore** | **Clone** | **Vendor** | **Catalog Number** | **Dilution** | **Antigen retrieval buffer** | |
| --- | --- | --- | --- | --- | --- | --- | --- | --- |
| **Primary antibodies** | | | | | | | |  |
| EpCAM | Mouse | BV421 | 9C4 | BioLegend | 324220 | 1:40 (FC) | n/a | |
| HTII-280 | Mouse | unconjugated | TB-27AHT2-280 | Terrace Biotech | TB-27AHT2-280 | 1:40 (FC), 1:50 (IF) | n/a | |
| TTF1/NKX2-1 | Mouse | unconjugated | 8G7G3/1 | Santa Cruz Biotechnology | Sc-53136 | 1:50 | n/a | |
| KRT5 | Rabbit | unconjugated | Poly19055 | BioLegend | 905501 | 1:100 | n/a | |
| SFTPC | Rabbit | unconjugated | polyclonal | Millipore Sigma | AB3786 | 1:50 | Diva | |
| ACE2 | Rabbit | unconjugated | polyclonal | Abcam | Ab15348 | 1:200 | Borg | |
| AGER | Goat | unconjugated | Polyclonal | R&D Systems | AF1145 | 1:500 | Diva | |
| SCGB1A1 | Mouse | unconjugated | E-11 | Santa Cruz Biotechnology | sc-365992 | 1:500 | Diva | |
| AcTub | Mouse | unconjugated | 6-11B-1 | Millipore Sigma | T7451 | 1:100 | Diva | |
| **Secondary antibodies** | | | | | | | |  |
| Mouse IgM | Rat | FITC | II/41 | Invitrogen | 11-5790-81 | 1:100 (FC), 1:300 (IF) |  | |
| Mouse IgG | Donkey | AlexaFluor488 |  | Thermo Fisher Scientific | A-21202 | 1:300 |  | |
| Rabbit IgG | Donkey | AlexaFluor568 |  | Thermo Fisher Scientific | A10042 | 1:300 |  | |
| Goat IgG | Donkey | AlexaFluor647 |  | Thermo Fisher Scientific | A-21447 | 1:300 |  | |

**Supplementary Table 4. Primer sequences used for q-RT-PCR**

| **Primer** | **Sequence (5’-3’)** |
| --- | --- |
| SARS-CoV-2 E sgRNA | (F) CGATCTCTTGTAGATCTGTTCTC, (R) ATATTGCAGCAGTACGCACACA, (P) ACACTAGCCATCCTTACTGCGCTTCG |
| SARS-CoV-2 E gRNA | (F) ACAGGTACGTTAATAGTTAATAGCGT, (R) ATATTGCAGCAGTACGCACACA, (P) ACACTAGCCATCCTTACTGCGCTTCG |
| Human GAPDH | (F) GAAGGTGAAGGTCGGAGTCAAC, (R) CAGAGTTAAAAGCAGCCCTGGT, (P) TTTGGTCGTATTGGGCGCCT |
| Human ACTB | (F) TCACCCACACTGTGCCCATCTACGA, (R) CAGCGGAACCGCTCATTGCCAATGG, (P) ATGCCCTCCCCCATGCCATCCTGCGT |
| Human ISG15 | (F) GCCTTCAGCTCTGACACC, (R)  CGAACTCATCTTTGCCAGTACA, (P)  CACCTGGAATTCGTTGCCCGC |
| Human ISG20 | (F) CTCGCATCTTCCACCGA, (R) AATCTACGACACGTCCACTG, (P) TCACTCAGCACCCGCAGGG |
| Human IFITM3 | (F) AAAGCGTGTGAGGATAAAGGG, (R)  AGGCCTATGGATAGATCAGGAG, (P)  CGAGGAATGGAAGTTGGAGTACGTGG |

**
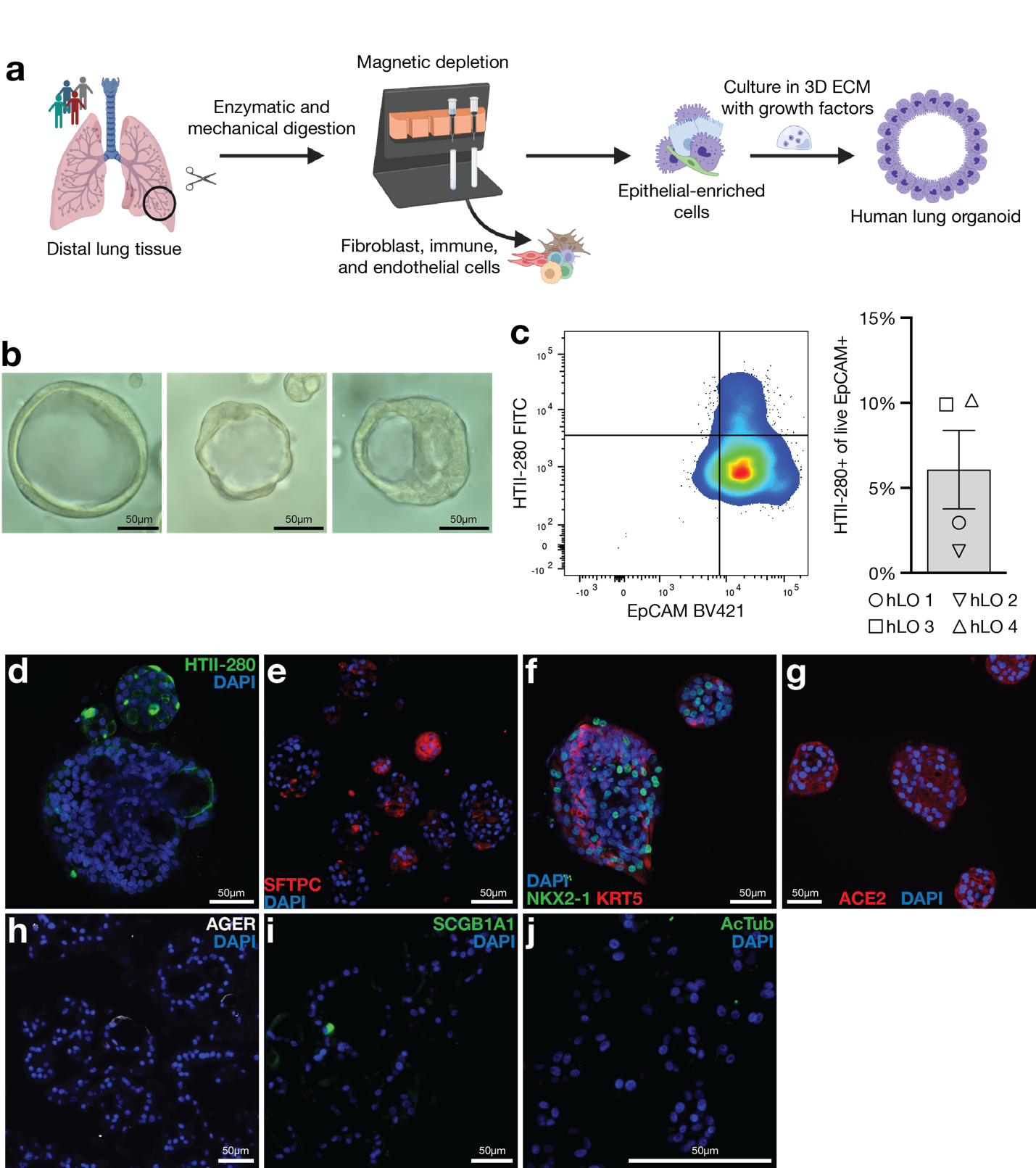
**

**Supplementary figure 1. Characterization of hLOs.** (a) Procedure used to generate hLOs from distal lung tissue. (b) Brightfield microscopy images of hLOs in culture. (c) hLOs were analyzed by flow cytometry for expression of EpCAM and HTII-280. Intact, singlet, live cells are shown. hLOs were analyzed by immunofluorescence microscopy for expression of HTII-280 (d), SFTPC (e), NKX2-1 and KRT5 (f), ACE2 (g), AGER (h), SCGB1A1 (i), or AcTub (j). Immunostaining was done on FFPE sections and images acquired by widefield microscopy (e, g-j) or whole-mount staining followed by confocal microscopy with maximum intensity projection (d, f).

**
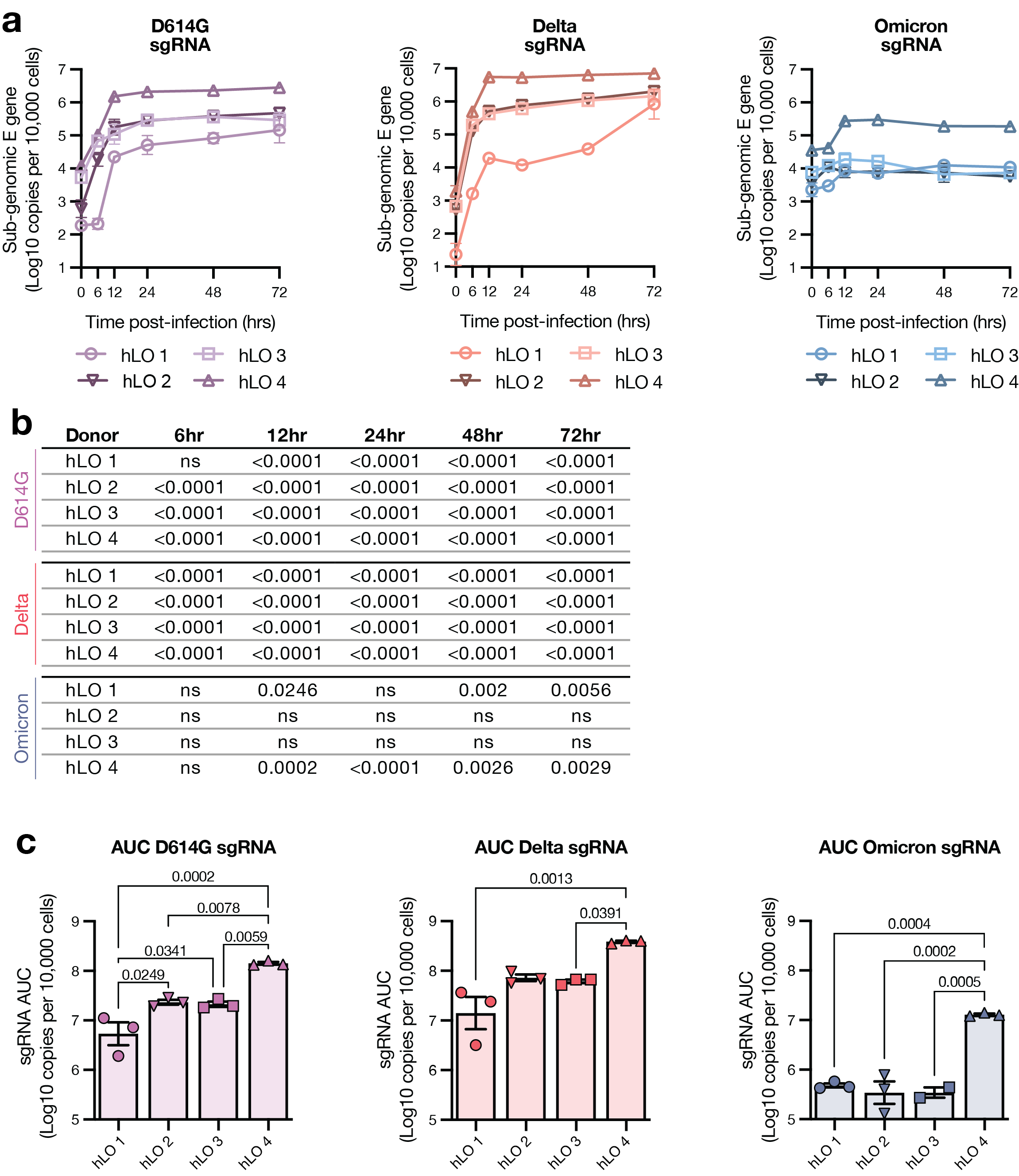
**

**Supplementary figure 2. Variable viral load in hLOs derived from different donors.** (a) sgRNA viral load was measured by qRT-PCR in hLOs from each donor inoculated with D614G, Delta, or Omicron. (b) Statistical analysis of sgRNA viral load as shown over time for each donor compared to 0hr post-infection. Statistical analysis was performed using two-way ANOVA with Dunnett’s post-test. Ns: not statistically significant (P>0.05). (c) sgRNA copy numbers over time were used to calculate AUC relative to RNA copies at 0hr post-infection for each donor. Mean +/- SEM of n=3 replicates per donor hLO are shown. Statistical analysis was performed using one-way ANOVA with Tukey’s post test (c). P-values < 0.05 are shown.

**
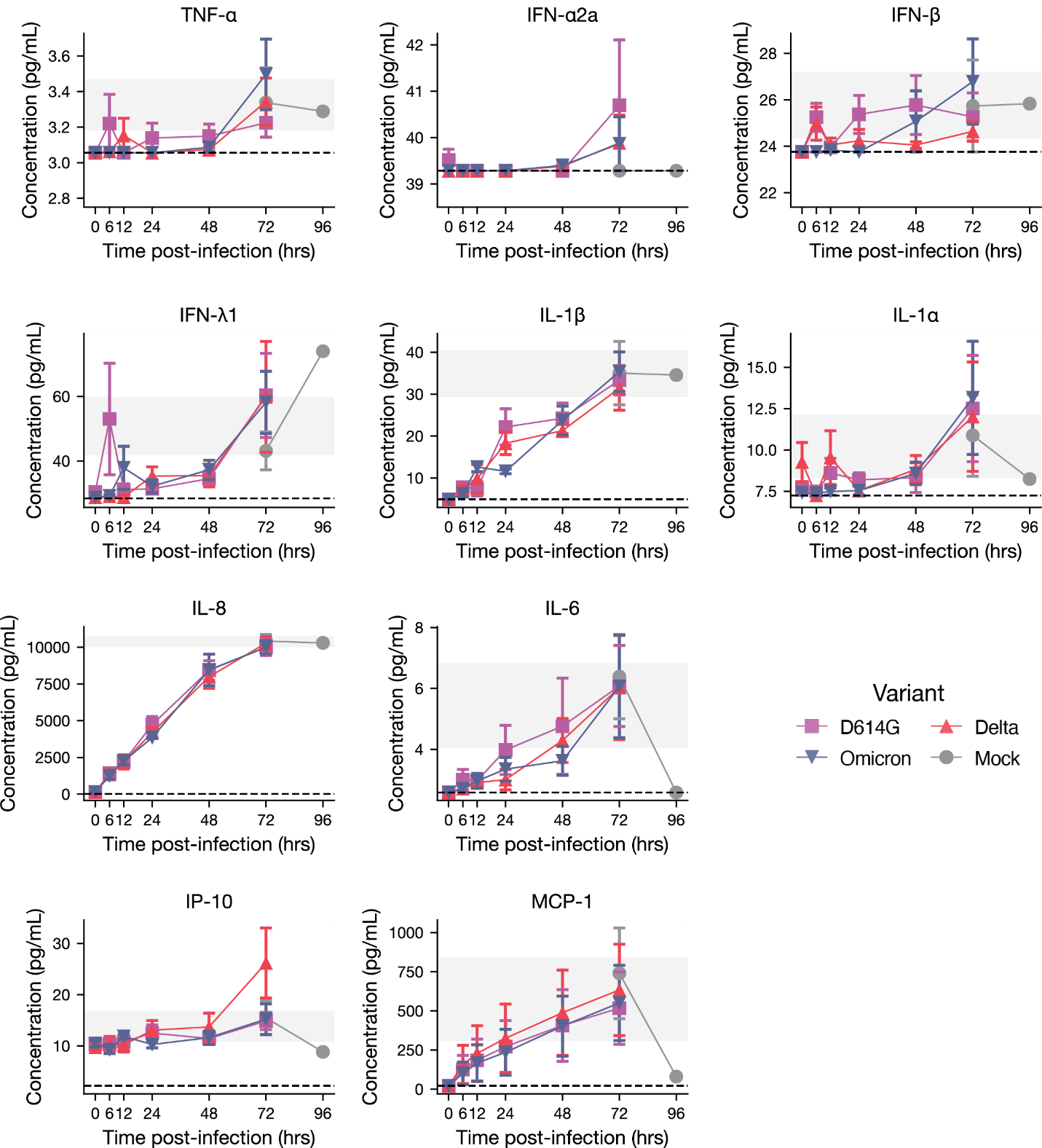
**

**Supplementary figure 3. hLOs do not secrete cytokines in response to SARS-CoV-2 infection.** hLO culture supernatants were analyzed for the presence of ten cytokines and chemokines at multiple timepoints post-infection. Supernatants from mock-infected control samples were collected at 72 hours post-infection (hLO 1-3) or 96 hours post-infection (hLO 4). Light gray shading denotes mean +/- SEM of mock-infected controls. Dashed line indicates lower limit of detection.

**
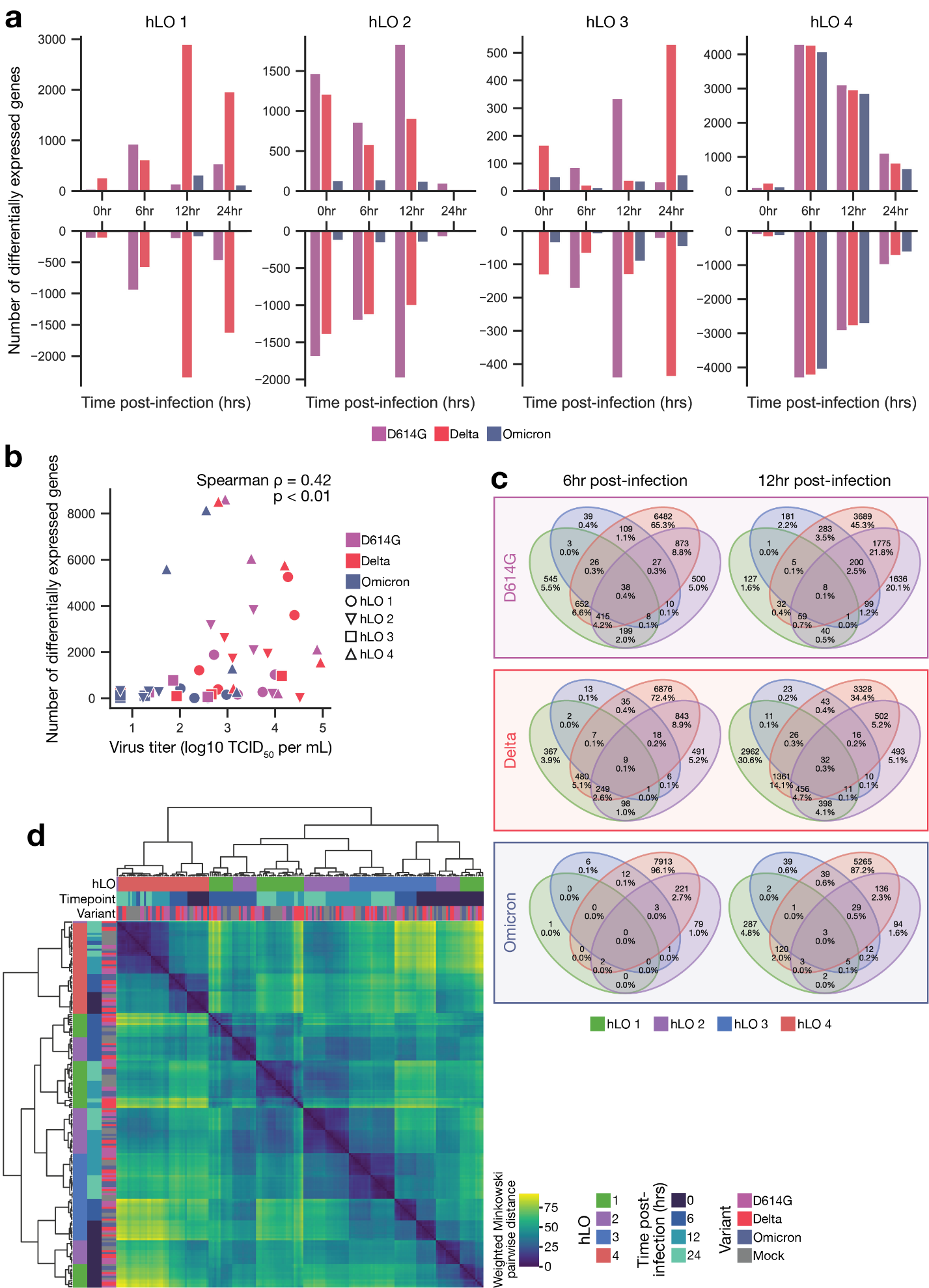
**

**Supplementary figure 4. Divergent transcriptional responses to SARS-CoV-2 infection in hLOs from different donors.** (a) The number of significantly differentially expressed genes (adjusted p-value < 0.1) versus mock-infected controls are plotted for each hLO donor, SARS-CoV-2 variant, and timepoint. (b) Spearman rank correlation between the number of significantly differentially expressed genes (as in a), and virus titer from matched samples. (c) Four-way venn diagrams illustrating the overlap (number and percentage) of differentially expressed genes (adjusted p-value < 0.1) between hLO donors at 6 and 12 hours post-infection. (d) A weighted Minkowski pairwise distance matrix was calculated on PCA-transformed data using the percentage of variance explained by each PC as weights. Samples were hierarchically clustered according to pairwise distances.

**
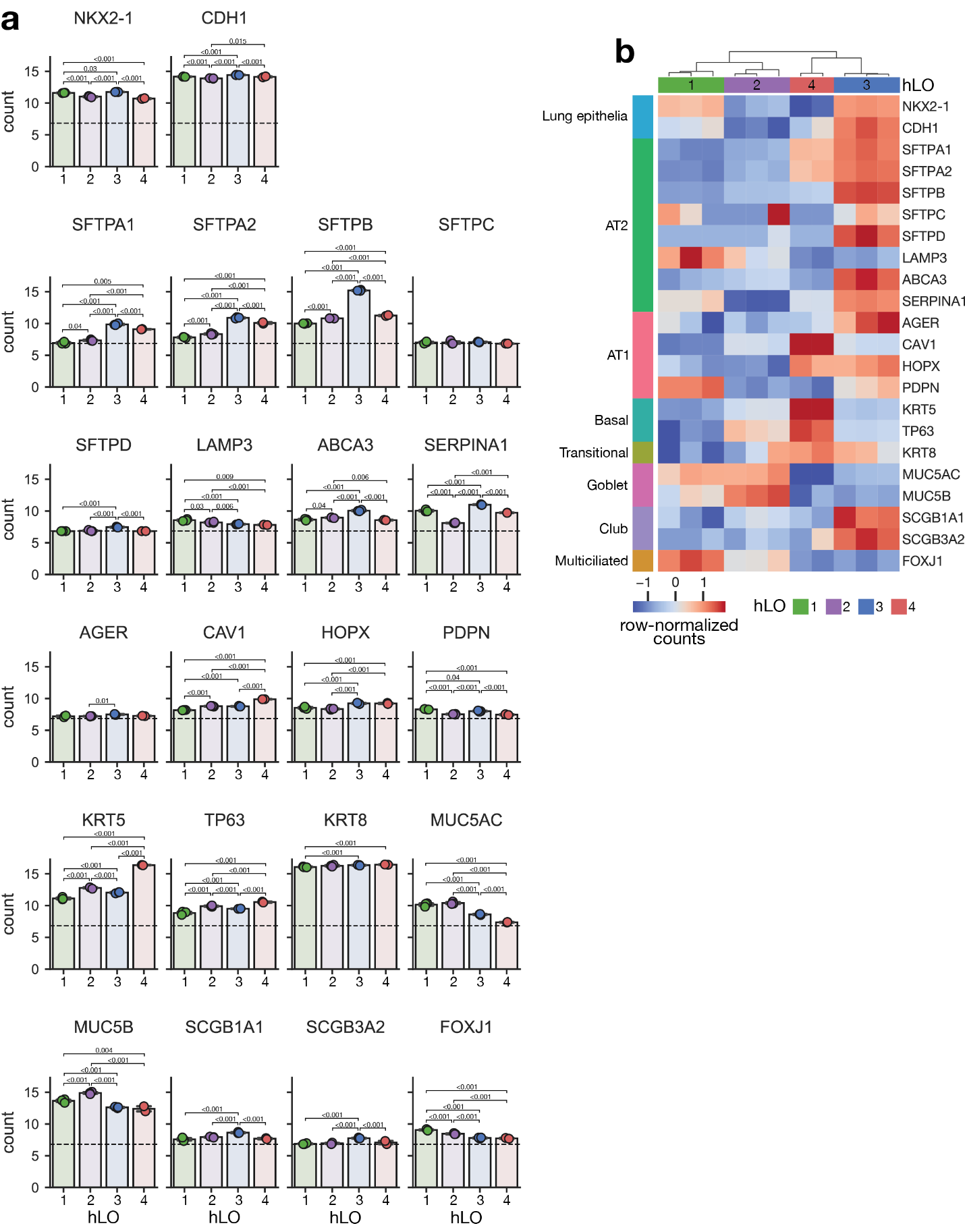
**

**Supplementary figure 5.** **Expression of canonical lung epithelial cell markers in hLOs.** (a) VST-normalized counts in uninfected samples of cell subset marker genes are shown. Statistical analysis was conducted using the Wald test with Benjamini-Hochberg adjustment for multiple test correction, as implemented in DESeq2. P-values < 0.05 are shown. (b) Uninfected samples from each hLO donor were hierarchically clustered according to their expression of the cell subset marker genes in a. Cell type subsets identified by the different markers displayed in a and b are indicated to the left of panel b.


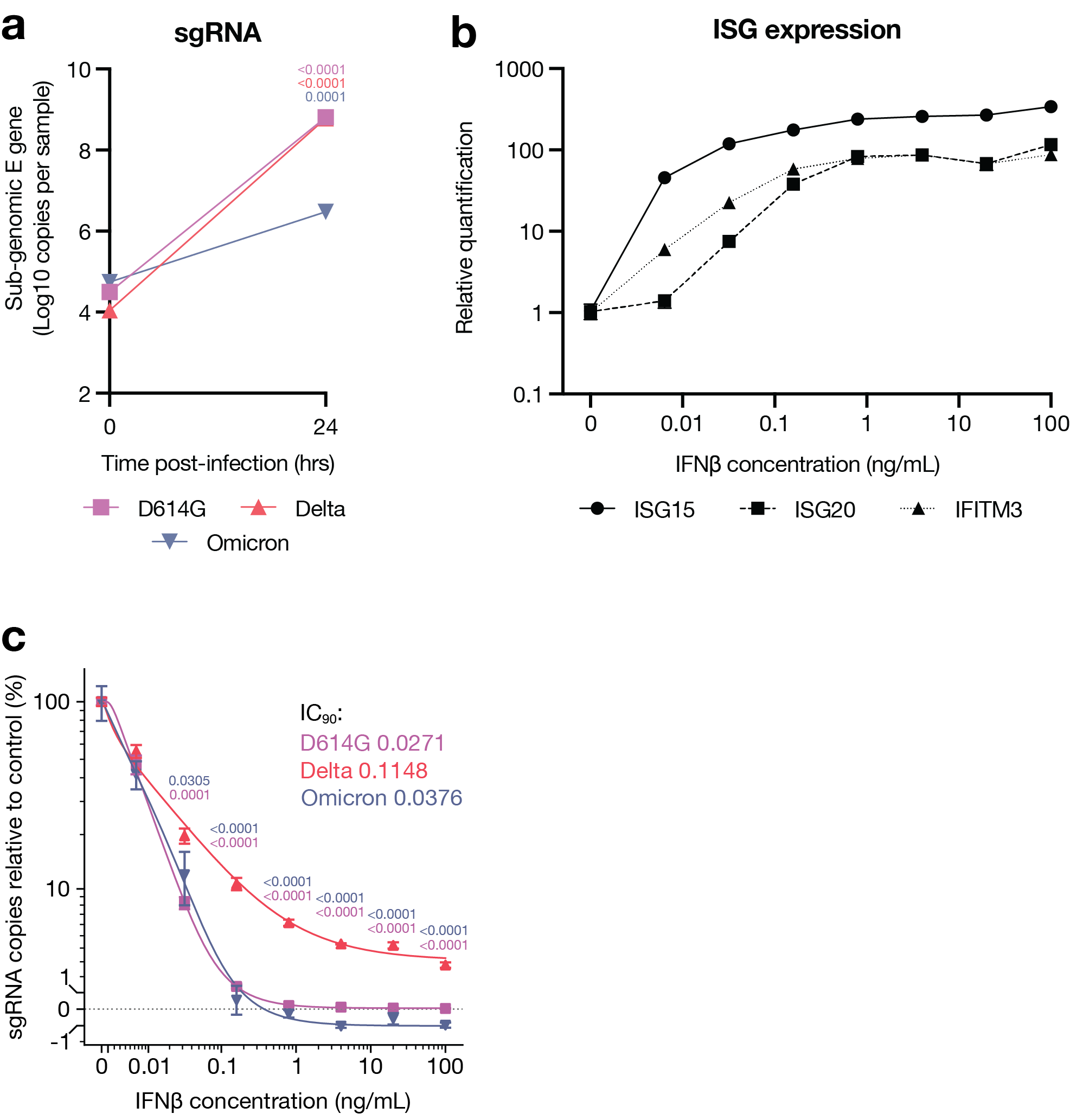


**Supplementary figure 6. IFN sensitivity of SARS-CoV-2 VOCs.** A549 AAT cells were treated with escalating doses of recombinant human IFNβ for 12 hours prior to infection with SARS-CoV-2 VOCs at MOI 0.1. (a) sgRNA viral load measured by qRT-PCR in untreated control samples (no IFNβ). Statistical analysis was performed for each variant using Student’s T test. P-values < 0.05 comparing 24hr vs 0hr post-infection are shown. (b) Dose-dependent ISG response at 0hr in mock-infected samples. (c) sgRNA copies at 24hr post-infection were normalized to a percentage of untreated controls after subtraction of input (0hr) sgRNA copies. Two-way ANOVA was conducted on log-normalized data. P-values represent Dunnett’s post-test of the indicated VOC vs Delta. IC90 was calculated using five-parameter logistic regression curves fit separately for each variant.
